# Genomic characterization of pre‐Green Revolution wheat landraces from the Nepalese and Bhutanese Himalayas reveals geographically structured diversity

**DOI:** 10.1002/tpg2.70320

**Published:** 2026-10-12

**Authors:** Li Yunfei, Meng Zhaojie, Miyuki Nitta, Atsushi Ohta, Kazuhiro Nemoto, Giri Prasad Joshi, Su Zhuo, Yasir Serag Alnor Gorafi, Shuhei Nasuda

**Affiliations:** ^1^ Graduate School of Agriculture Kyoto University Kyoto Japan; ^2^ Faculty of Agriculture Shinshu University Nagano Japan; ^3^ Central Department of Botany Tribhuvan University Kathmandu Nepal

## Abstract

Wheat (*Triticum aestivum* L.) landraces collected before the widespread regional adoption of Green Revolution cultivars provide a historical record of locally maintained germplasm. We characterized a panel of 202 accessions collected from Nepal and Bhutan between 1952 and 1958. Genotyping by Random Amplicon Sequencing‐Direct yielded 30,228 high‐quality markers; removing 38 close genetic duplicates produced a nonredundant panel of 164 accessions. We investigated genetic diversity, linkage disequilibrium, population structure, and spatial variation in effective migration, compared the panel with 187 analyzed accessions from a global hexaploid wheat core collection, and evaluated four agro‐morphological traits in a common environment. We identified eight geographically patterned genetic groups and 28 admixed accessions. All four traits differed significantly among groups, and plant height, days to heading, and days to flowering correlated with collection‐site altitude. The global core panel had higher overall marker‐based diversity. Nevertheless, Group 7, the Bhutan‐enriched Group 8, and several transitional accessions occupied regions of genetic space with limited representation in that panel. This historical collection therefore complements global core resources by providing fine‐scale regional diversity and a basis for accession selection for multi‐environment phenotyping, allele discovery, and pre‐breeding.

## Plain Language Summary

Wheat landraces collected in Nepal and Bhutan between 1952 and 1958 provide a historical sample of Himalayan germplasm from before the widespread adoption of Green Revolution cultivars. We genotyped all 202 accessions in this preserved collection and retained 164 after removing close genetic duplicates. The remaining accessions formed genetic groups associated with collection region and elevation, and four plant traits measured in one common field environment differed among groups. A global wheat comparison panel had greater overall genetic diversity, but several Himalayan groups were poorly represented. The study does not directly demonstrate genetic erosion, climate resilience, or adaptive genes. Instead, it identifies differentiated accessions for future testing across environments to validate their utility for use in crop improvement.

**Abbreviations**
ANOVA: analysis of variance
CV: cross‐validation
DST: IBS distances
DTF: days to flowering
DTH: days to heading
EAA: environmental association analyses
ETN: effective number of tillers
FEEMS: Fast and Flexible Estimation of Effective Migration Surfaces
GBS: genotyping‐by‐sequencing
GRAS‐Di: Genotyping by Random Amplicon Sequencing‐Direct
IBS: identical‐by‐state
INDELs: insertions/deletions
LD: linkage disequilibrium
NBRP‐Wheat: National BioResource Project‐Wheat
PCA: principal component analysis
PH: plant height
PIC: polymorphism information content
SNP: single‐nucleotide polymorphism
SVs: structural variations
VCF: variant call format
WGS: whole‐genome sequencing

## 1 INTRODUCTION

Wheat (*Triticum aestivum* L.) is among the most extensively cultivated and consumed cereal crops worldwide, serving as a critical source of dietary minerals, sugars, and proteins (Shewry & Hey, 2015). However, ensuring future wheat supply poses considerable challenges. By 2050, the global population is expected to surpass 9 billion, necessitating substantial increases in crop productivity (van Dijk et al., 2021). Concurrently, climate variability—manifested through heatwaves, droughts, and altered precipitation patterns—threatens wheat production (B. Liu et al., 2016). A further constraint arises from the limited genetic diversity of modern elite wheat cultivars, a consequence of the intensive breeding strategies employed during the Green Revolution (Evenson & Gollin, 2003; Wouw et al., 2010). Although this period achieved remarkable yield improvements, it also contributed to genetic erosion by favoring a genetically narrow set of elite high‐yielding varieties (Wouw et al., 2010). To sustain progress in wheat improvement, it is imperative to harness the extensive, underutilized genetic diversity found in landraces (Cheng et al., 2024). These landraces represent rich reservoirs of alleles conferring resistance to biotic and abiotic stresses. Notably, two putative loci associated with yellow rust resistance were recently identified in Himalayan wheat accessions (Jung et al., 2025), providing an example of the potential value of this regional germplasm.

The Himalayan region, characterized by steep altitudinal gradients ranging from subtropical plains to alpine highlands, encompasses a mosaic of microclimates that offer a unique context for studying plant genetic differentiation across geography and elevation (Körner, 2007; Sun et al., 2025). Wheat cultivation in this region spans millennia, exposing landraces to diverse and intense selective pressures. Despite their adaptive significance, Himalayan wheat landraces—particularly those from Nepal and Bhutan—remain underrepresented in global germplasm repositories, including the Watkins collection (Wingen et al., 2014). The genetic structure of these regional landraces and their contribution to broader wheat diversity therefore remain incompletely characterized.

The panel examined here comprises 202 hexaploid wheat accessions collected in Nepal and Bhutan during Japanese expeditions in 1952 and 1953 (Nakao & Fauna & Flora Research Society, 1955) and in 1958, with the latter expedition documented in the Nakao Sasuke Slide Database (Osaka Metropolitan University, n.d.). These accessions are currently maintained as part of the hexaploid wheat germplasm collection of the National BioResource Project‐Wheat (NBRP‐Wheat), Japan (Takenaka et al., 2019). The collection sites span the southern flank of the Himalayas and range from ∼400 to 3900 m, including lowland plains, river valleys, and mountain slopes. Although the expeditions did not sample Nepal and Bhutan exhaustively, the resulting collection covers a substantial geographical and elevational range. The collection's historical value is reinforced by its collection dates, which precede the widespread regional adoption of Green Revolution cultivars. For brevity, we refer to these accessions hereafter as the “Himalayan wheat landrace panel.”

Based on the uniqueness of this population's geographical distribution and collection time, we hypothesized that (1) the Himalayan panel would show geographically structured genetic differentiation accompanied by admixture, (2) genetic groups associated with contrasting collection‐site elevations would differ in agro‐morphological traits, and (3) this landrace collection might not be well represented in the NBRP global hexaploid wheat core collection (Takenaka et al., 2019). Accordingly, we aimed to characterize genetic diversity and population structure within the Himalayan panel, relate the inferred genetic groups to collection geography, elevation, and four agro‐morphological traits, and evaluate their representation in the global core collection.

## 2 MATERIALS AND METHODS

### 2.1 Plant materials

The plant materials comprised 202 hexaploid wheat accessions currently maintained as part of the germplasm collection of the NBRP‐Wheat, Japan (Takenaka et al., 2019). These accessions were all the available ones collected in Nepal and Bhutan during Japanese expeditions in 1952, 1953, and 1958 (see Table S1 for detailed passport data). Among the 202 accessions, 172 were collected from Nepal and 30 from Bhutan. This Himalayan wheat landrace panel demonstrates broad geographical provenance spanning the southern flank of the Himalayan range, encompassing altitudinal gradients from 400 to 3900 m above sea level across lowland plains, river valleys, and high‐altitude slopes (Figure 1A; Table S1). After identification and removal of duplicate accessions (as detailed in Section 2.3), 38 accessions were removed and 164 unique ones were retained for downstream genomic and phenotypic analyses.

**FIGURE 1:**
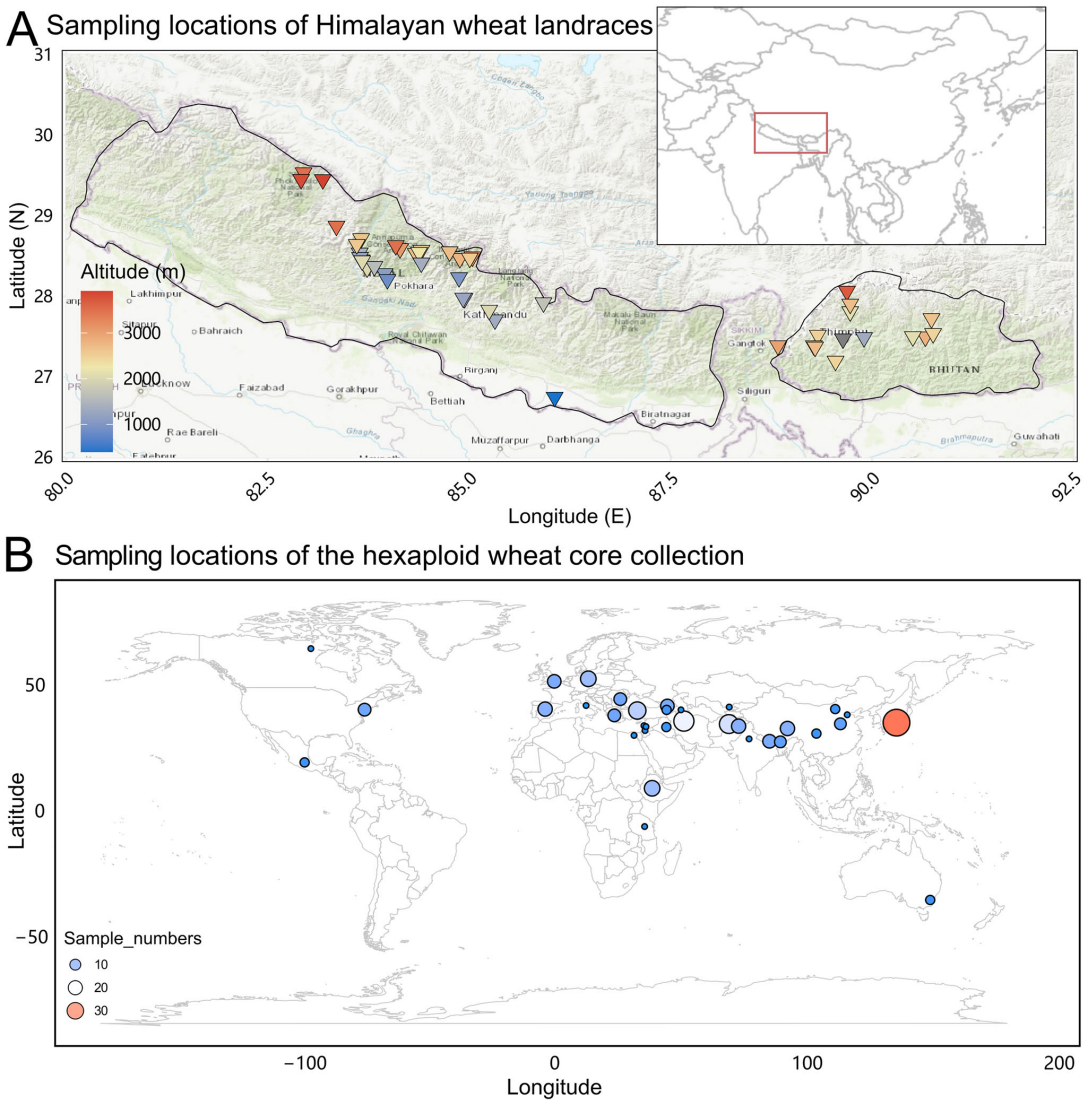
Geographical distribution of Himalayan wheat landraces and the hexaploid wheat core collection. (A) Collection sites of Himalayan wheat landraces. Each triangle represents one collection site; note that more than one accession may be collected at a site. (B) Geographical distribution of hexaploid wheat core collection.

To assess the extent to which the diversity of the Himalayan wheat is captured in current wheat core collection panels, we compared the current population with the NBRP global hexaploid wheat core collection (Takenaka et al., 2019) (see sample list in Table S2). The hexaploid wheat core collection comprises 188 accessions collected from over 30 countries/regions worldwide. It represents five continental zones: Asia, Europe, Africa, North America, and Oceania (Figure 1B). It was genotyped using the Genotyping by Random Amplicon Sequencing‐Direct (GRAS‐Di) strategy (Su et al., 2026).

### 2.2 DNA extraction and genotyping

For genomic DNA extraction, five seeds from each of the 202 accessions were directly germinated in cell boxes. Two healthy, homogeneous seedlings from each accession were transferred to cell trays. Around 10 days after germination, at the second leaf stage, we collected tender leaves from two individuals per accession and immediately fixed them in liquid nitrogen for DNA extraction. Our extraction protocol follows Takenaka et al. (2019). DNA concentration, purity, and integrity were checked with a NanoDrop 2000 Spectrophotometer and 1% agarose gel electrophoresis. Subsequently, DNA extracts were sent to Toyota Motors Company for GRAS‐Di (T. Enoki & Drake, 2017; Dukamo et al., 2024; H. Enoki & Takeuchi, 2018), an advanced genotyping‐by‐sequencing (GBS) platform. GRAS‐Di enables the generation of thousands of high‐quality markers distributed across the wheat genome, including centromeric regions that are typically underrepresented in other GBS approaches (Fekih et al., 2023; H. Enoki &, Takeuchi, 2018).

Raw reads from the current panel were combined with the NBRP hexaploid wheat core collection for variant calling and subsequent analysis. Sample 6 × 099 was excluded from subsequent analysis due to its low sequencing depth (see Table S3).

Quality control of raw reads was performed using FastQC v0.12.1 (Babraham Bioinformatics, n.d.), MultiQC v1.25.1 (Ewels et al., 2016), and fastp v0.23.4 (S. Chen, 2023) with default parameters. We then mapped the clean reads using bwa mem v0.7.18‐r1243‐dirty (Vasimuddin et al., 2019), with the IWGSC Chinese Spring Ref v2.1 as the reference genome (Zhu et al., 2021). Variant calling was performed using a joint calling pipeline with GATK4 v4.5.0.0 (Auwera & O'Connor, 2020) and samtools v1.21 (Danecek et al., 2021), following the optimum practice for semantic variant discovery. In short, the steps (and tools) in our variant calling pipeline involved mark duplicates (*gatk MarkDuplicates*), haplotype calling (*gatk HaplotypeCaller*), and joint calling (*gatk CombineGVCFs* and *gatk GenotypeGVCFs*). The intermediate files in SAM or BAM format were processed with samtools (Li et al., 2009). The resulting variants for the two populations were stored in a single variant call format (VCF) file.

For variant filtering, a two‐step strategy was applied. In the first step, we split the VCF file using *gatk SelectVariants* into two subsets: one containing only single‐nucleotide polymorphisms (SNPs) and the other containing only insertions/deletions (INDELs). Because bread wheat is a hexaploid species with high homoeology among its subgenomes, stringent filtering was imperative to ensure subgenome specificity and eliminate false polymorphisms arising from homoeologous sequence variations. Specifically, multimapping reads were rigorously excluded by applying a mapping quality (MQ) threshold of <40.0. To further ensure specific alignment, we applied an MQRankSum < −12.5, which penalizes sites where the MQ of reads supporting the alternative allele is lower than that of reads supporting the reference allele—a common signature of misaligned homoeologous reads (Su et al., 2026). SNP variants were ultimately filtered with “QD [QualByDepth] < 2.0, QUAL [Variant Quality Score] < 30.0, SOR [StrandOddsRatio] > 4.0, FS [FisherStrand] > 60.0, MQ < 40.0, MQRankSum < −12.5, and ReadPosRankSum < −32.0.” INDEL variants were filtered with “QD < 2.0, QUAL < 30.0, SOR > 10.0, FS > 200.0, MQ < 40.0, MQRankSum < −20.0.” The primarily filtered SNP and INDEL variants were then merged into a single VCF file using *gatk MergeVcfs*. In the second step, all heterozygous sites were removed with bcftools v1.21 (Danecek et al., 2021), assuming they were erroneous, since all accessions were maintained through self‐pollination for multiple generations. Afterward, the variants were split into two populations, and a missing rate <20% was used to filter each population in *TASSEL 5* (Bradbury et al., 2007). Then, two VCF files were merged, and minor allele frequency > 0.01 was set to remove rare alleles (TASSEL 5).

After filtration, the density maps of the remaining markers were plotted using the CMplot v4.5.1 (LiLin‐Yin, 2024) package in *R v4.4.1* (R Core Team, 2024).

### 2.3 Genetic diversity and population structure analysis

To minimize potential bias from physically linked markers in relatedness and population structure estimates, the high‐quality SNP dataset was first pruned for linkage disequilibrium (LD) using PLINK v1.9.0‐b.8 (Purcell et al., 2007). The pruning parameters were set to a sliding window of 50 SNPs, a step size of five SNPs, and an *r* ^2^ threshold of 0.2 (–indep‐pairwise 50 5 0.2). To mitigate potential bias in downstream analyses caused by highly related or duplicated individuals, we calculated pairwise identical‐by‐state (IBS) similarity based on the LD‐pruned dataset using PLINK. Based on the distribution of IBS distances (DST) values and community conventions, accessions sharing a DST > 0.98 were considered duplicates, and in each duplicated group, the accession with lowest missing rate was kept (Figure S1). This filtering step identified and removed 38 duplicated accessions, leaving 164 accessions for further analysis.

For genetic diversity statistics, we deployed TASSEL 5 to calculate nucleotide diversity (*π*) per site, Watterson's Theta (*θ*) per site at each chromosome, subgenome, and the whole genome, and LD, using all available high‐quality markers. Additionally, expected heterozygosity (*H_e_*) and polymorphism information content (PIC) were calculated using *vcftools* (Danecek et al., 2011) and custom *R* scripts. LD was measured as the allele‐frequency correlation (*r* ^2^) for all pairwise SNP comparisons on each chromosome, and the chromosome‐ and genome‐specific mean values were subsequently estimated. Interchromosomal LD was estimated over the whole genome. LD decay was estimated separately for the Himalayan and NBRP global core panels using the same marker filters and analytical procedure. Pairwise LD was calculated in TASSEL 5 using a sliding window of 50 SNPs. Decay curves were fitted in R using the nonlinear expectation of *r* ^2^ described by Hill and Weir (1988) and implemented following Remington et al. (2001) and Roncallo et al. (2021). Pairwise LD values were down‐sampled only for visualization, whereas all valid observations were retained for model fitting and decay‐threshold estimation. For population structure analysis, a pairwise genetic distance matrix and principal component analysis (PCA) were performed with TASSEL 5 using the LD‐pruned dataset. After pruning with PLINK, 5573 and 9336 markers were left for the Himalayan collection and hexaploid wheat core collection. Following this, the pairwise genetic distance matrix was used for hierarchical clustering with the *R* function *hclust* using the “complete” method. IQ‐TREE was used to identify the best‐fitting model and construct the maximum‐likelihood phylogenetic tree (Minh et al., 2020). Detailed population structure was estimated using ADMIXTURE (Alexander et al., 2009) based on LD‐pruned SNPs, with the number of ancestral populations (*K*) ranging from 2 to 20 and 10 cross‐validations (CVs). Although *K* = 10 yielded the lowest CV error, *K* = 8 represented the first local minimum, and the reduction in CV error from *K* = 8 to *K* = 10 was marginal. We therefore selected *K* = 8 as a parsimonious representation of the major ancestry structure and to avoid over partitioning. At *K* = 8, accessions with a maximum ancestry proportion (*Q*_max_) ≥ 0.70 were assigned to the corresponding genetic group, whereas accessions with *Q*_max_ < 0.70 were classified as admixed. The output subpopulation matrices were aligned in the order of each accession on the IQ‐TREE output. To assess the degree of genetic differentiation among the identified subpopulations, pairwise fixation index (*F*_st_) values were also computed with *vcftools*.

To estimate the inferred relative effective migration between different accessions, Fast and Flexible Estimation of Effective Migration Surfaces (FEEMS) was used to calculate and visualize the immigration pattern (Marcus et al., 2021).

### 2.4 Field plot design and phenotyping

Field phenotyping accessions were planted at the Kyoto University Experimental Farm (N35.032, E135.784) in a single‐row design during the 2023–2024 planting season. For each accession, five seeds were germinated in cell boxes on December 5, 2023, and transferred into cell trays 3 days later and allowed to grow under a polytunnel until the second leaf stage. Afterward, they were transplanted into the field on December 21, 2023. Three individuals were grown in the field unless they failed to survive until the transplant day, with ∼25 cm spacing between individuals.

When the plants reached the heading and flowering stages, the specific dates were recorded as heading and flowering dates, then used to calculate days to heading (DTH) and days to flowering (DTF). The heading and flowering stages were characterized according to Zadoks’ stages 59 and 61, respectively (Zadoks et al., 1974). DTH and DTF were calculated as the difference between observed heading/flowering dates and germination date. At harvest time, we removed each plant from the soil and measured plant height (PH) and counted its effective number of tillers (ETNs). ETN was defined as the number of tillers that successfully develop into productive spikes (J. Liu et al., 2020). The average values for DTH, DTF, PH, and ETN across all available individuals were used for subsequent analysis.

All statistical analyses were conducted in R v4.4.1 (R Core Team, 2024), and accession‐mean values were treated as independent observations. Trait distributions among the eight genetic groups and the admixed category were visualized using *tidyverse* v2.0.0 (Wickham et al., 2019). Differences among groups were evaluated separately for PH, DTH, DTF, and ETN using one‐way analysis of variance (ANOVA) implemented in *ggpubr v0.6.0* (Kassambara, 2023). When the overall group effect was significant, pairwise comparisons were conducted using Tukey's honestly significant difference test at *p* < 0.05. Spearman's rank correlations were used to evaluate associations between collection‐site altitude and accession‐mean trait values.

## 3 RESULTS

### 3.1 GRAS‐Di genotyping and duplicate filtering of the Himalayan wheat landrace panel

After removing low‐quality reads and trimming sequencing adapters, we retained, on average, 4.95 million clean reads per sample (Table S3). Mean GC (guanine‐cytosince) content and average length of the clean reads were 52% and 149.3 bp, respectively. After variant calling and filtering, 30,228 high‐quality markers were obtained. To address potential sample redundancy within these localized collections, we evaluated pairwise IBS DST among the 202 accessions. The distribution of pairwise DST values revealed the presence of highly related or duplicated individuals (Figure S1A). By applying a stringent cutoff of DST > 0.98, we identified and removed 38 duplicated accessions (Figure S1B). Consequently, a refined panel of 164 unique accessions, together with the 30,228 high‐quality markers, was retained for subsequent diversity and population structure analyses.

Marker distribution was not uniform across the subgenomes (Figure 2A), with the A, B, and D subgenomes containing 10,583, 14,511, and 5134 markers, respectively. At the chromosomal resolution, we observed large variation in polymorphism, with chromosomes 1D and 1B exhibiting the lowest and highest SNP counts (592 and 2349, respectively). Homoeologous group stratification revealed notable divergence, with Group 5 showing the lowest cumulative marker count (5A, 5B, and 5D; *Σ* = 3838) and Group 3 the highest (3A, 3B, and 3D; *Σ* = 4753). Notably, the obtained markers were well distributed across the genome and covered the centromere regions on each chromosome (Figure 2B).

**FIGURE 2:**
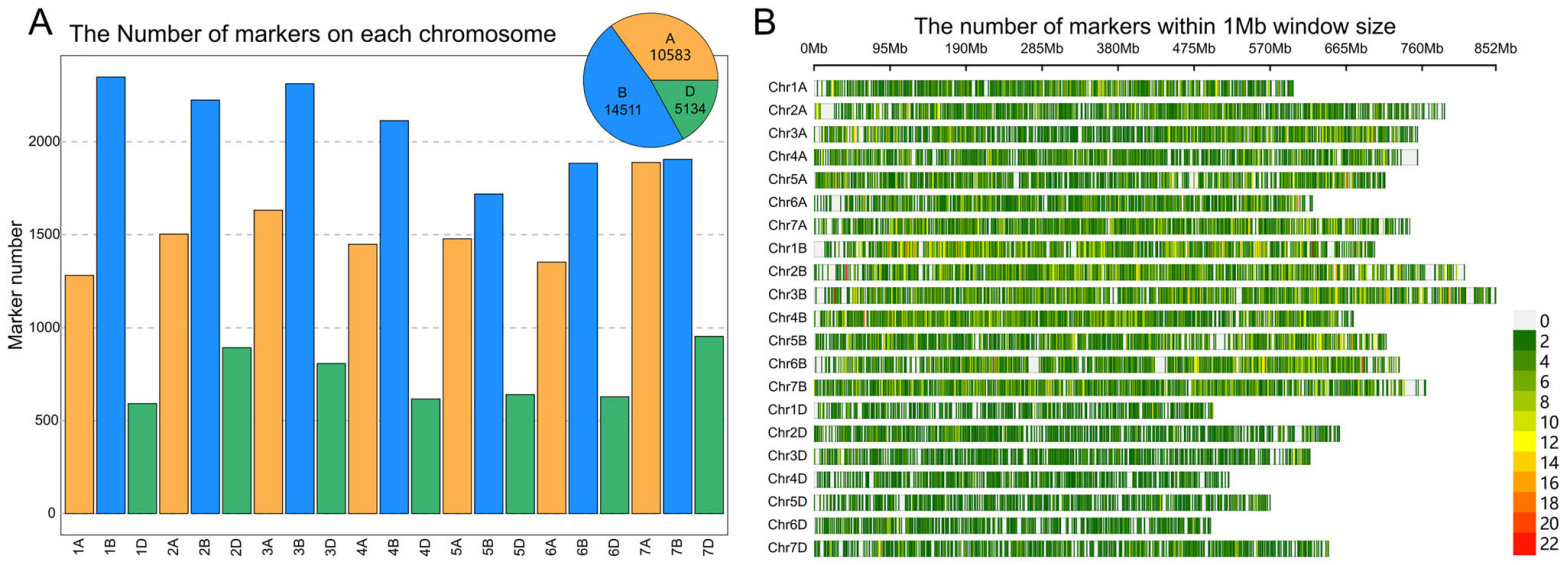
Summary of Genotyping by Random Amplicon Sequencing‐Direct (GRAS‐Di)‐based genotyping. (A) The total number of markers on each chromosome and subgenome; (B) the distribution and density of markers across each chromosome, visualized as the number of markers within each 1 Mb block.

### 3.2 Genetic diversity, LD decay pattern, and population structure of the Himalayan wheat landrace panel

To comprehensively characterize the genomic diversity of Himalayan wheat landraces, we performed multi‐scale analyses to calculate nucleotide diversity (*π*) and Watterson's Theta (*θ*), as well as *H_e_* and PIC at the chromosome, subgenome, and whole‐genome levels (Table 1). The Himalayan wheat landrace panel exhibited a genome‐wide average *π* value of 0.0666, an average *θ* value of 0.0856, a mean *H_e_* of 0.0821, and an average PIC of 0.0734. At the subgenome level, the respective *π* values for the A, B, and D subgenomes were 0.0681, 0.0697, and 0.0548, with corresponding *θ* values of 0.0868, 0.0800, and 0.0988. Furthermore, the *H_e_* values for the A, B, and D subgenomes were 0.0828, 0.0932, and 0.0551, with corresponding PIC values of 0.0739, 0.0828, and 0.0510, respectively.

**TABLE 1:** Genetic diversity of Himalayan wheat landraces and hexaploid wheat core collection.

Chromosome	Site	Polymorphism	Himalayan wheat landraces				Hexaploid core collection				
	count	site count	*π* per site	*θ* per site	*H_e_*	PIC	*π* per site	*θ* per site	*H_e_*	PIC
Chr_1A	1281	1180	0.1055	0.118	0.0962	0.0849	0.2372	0.1607	0.1119	0.1008
Chr_2A	1503	1411	0.0614	0.0692	0.092	0.0829	0.2131	0.1637	0.1429	0.1211
Chr_3A	1632	1474	0.0531	0.0708	0.0781	0.0704	0.2239	0.1575	0.0813	0.0734
Chr_4A	1448	1313	0.0799	0.1139	0.0739	0.0657	0.2424	0.158	0.1219	0.1087
Chr_5A	1478	1368	0.0783	0.0845	0.099	0.0879	0.2278	0.1614	0.0931	0.0834
Chr_6A	1352	1254	0.0374	0.0659	0.0564	0.0523	0.1825	0.1615	0.0894	0.0801
Chr_7A	1889	1806	0.0662	0.0892	0.0789	0.0702	0.2389	0.1671	0.0908	0.0817
Subgenome A	10,583	9806	0.0681	0.0868	0.0828	0.0739	0.2285	0.1616	0.1037	0.0921
Chr_1B	2349	2242	0.0497	0.0677	0.0758	0.0685	0.2246	0.1666	0.0856	0.077
Chr_2B	2225	2066	0.0596	0.0811	0.0763	0.0689	0.2237	0.162	0.0966	0.0867
Chr_3B	2313	2170	0.0668	0.0799	0.0876	0.0785	0.2122	0.1636	0.1226	0.1082
Chr_4B	2114	1987	0.0398	0.0559	0.0762	0.068	0.2503	0.1635	0.0571	0.0532
Chr_5B	1719	1589	0.0639	0.0878	0.0772	0.0687	0.2441	0.1614	0.1191	0.1047
Chr_6B	1885	1762	0.0982	0.0985	0.1058	0.0941	0.2197	0.1632	0.1215	0.107
Chr_7B	1906	1781	0.1207	0.096	0.1423	0.1231	0.2704	0.1627	0.1087	0.0969
Subgenome B	14,511	13,597	0.0697	0.08	0.0932	0.0828	0.2357	0.1634	0.1006	0.0897
Chr_1D	592	499	0.0555	0.1092	0.0501	0.0464	0.2134	0.1467	0.053	0.0496
Chr_2D	893	746	0.0518	0.0913	0.0566	0.0523	0.1945	0.1459	0.0487	0.0464
Chr_3D	808	668	0.0666	0.1015	0.0663	0.0604	0.1988	0.1438	0.0441	0.0414
Chr_4D	617	480	0.053	0.1048	0.0487	0.0459	0.1767	0.1352	0.0318	0.0306
Chr_5D	641	548	0.0517	0.0989	0.0526	0.0486	0.2057	0.1487	0.0373	0.0355
Chr_6D	629	516	0.059	0.1052	0.0564	0.0517	0.2391	0.1425	0.0394	0.0367
Chr_7D	954	808	0.0479	0.0888	0.0526	0.0493	0.1683	0.1474	0.0493	0.0459
Subgenome D	5134	4265	0.0548	0.0988	0.0551	0.051	0.1969	0.1446	0.0441	0.0415
Whole genome	30,228	27,668	0.0666	0.0856	0.0821	0.0734	0.2286	0.1595	0.093	0.0831

Abbreviation: PIC, polymorphism information content.

Furthermore, we estimated the LD decay pattern using all available markers. As illustrated in Figure 3A, the genome‐wide LD in the Himalayan wheat landrace panel decayed to half of its maximum value at a physical distance of approximately 3.71 Mbp, *r* ^2^ = 0.2 at 4.79 Mbp, and to *r* ^2^ = 0.1 at approximately 13.75 Mbp.

**FIGURE 3:**
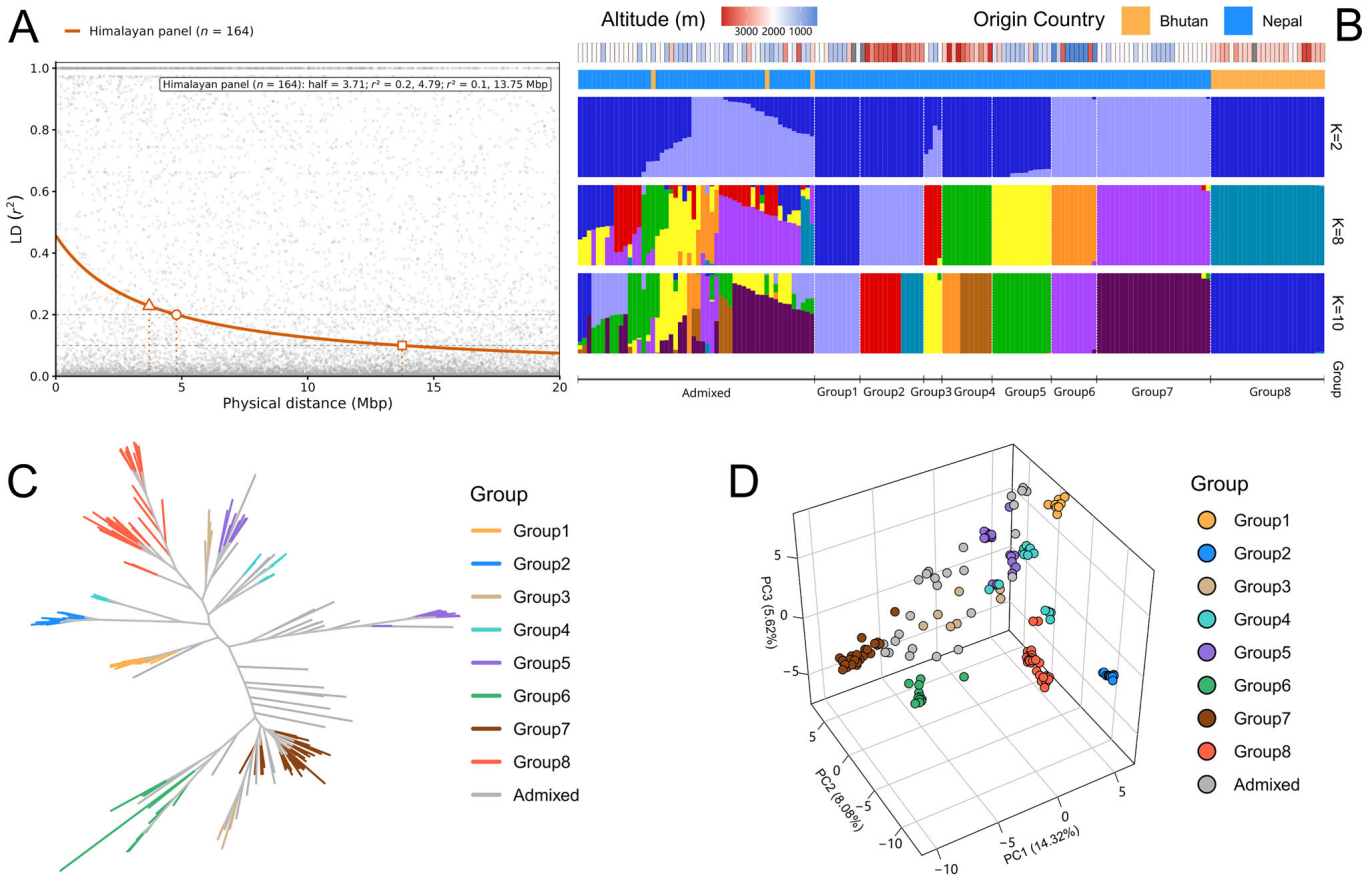
LD decay pattern and population structure analysis of Himalayan wheat landraces. (A) Genome‐wide linkage disequilibrium decay in the Himalayan panel (*n* = 164). The curve was fitted to all valid pairwise LD estimates using the Hill–Weir nonlinear model. Gray points represent a distance‐stratified subsample shown only for visualization; symbols indicate the fitted distances at half‐decay, *r^2^* = 0.2, and *r^2^* = 0.1. (B) Population structure inferred from pruned genotyping dataset with ADMIXTURE, at *K* = 2, *K* = 8, and *K* = 10. (C) Maximum likelihood phylogenetic tree, branch colored with subpopulations. (D) Principal component analysis (PCA) scores based on 5573 markers, colored with subpopulations.

To investigate population structure within the refined panel of 164 Himalayan wheat landraces, we analyzed 5573 SNP markers retained after LD pruning. The ADMIXTURE CV error was lowest at *K* = 10. However, the curve reached its first local minimum at *K* = 8, with only a marginal difference between the two values (Figure S2B). We therefore used *K* = 8 as the parsimonious grouping level for downstream analyses, while retaining *K* = 10 to illustrate finer‐scale ancestry patterns. At *K* = 8, 136 accessions with *Q*_max_ ≥ 0.70 were assigned to Groups 1–8, whereas the remaining 28 accessions were classified as admixed. We then compared these assignments with the maximum‐likelihood phylogeny, PCA, and pairwise genetic distance matrix and related them to accession geography and elevation (Figure 3B–D and Figures S2 and S3; Table S4).

Our analysis assigned eight genetic groups: Group 1 (*n* = 10), Group 2 (*n* = 14), Group 3 (*n* = 8), Group 4 (*n* = 14), Group 5 (*n* = 18), Group 6 (*n* = 12), Group 7 (*n* = 33), and Group 8 (*n* = 27), together with 28 admixed accessions. Groups 2, 6, and 8 showed the clearest correspondence between genetic assignment and collection geography: Group 2 occurred predominantly at northern high‐elevation sites, Group 6 at southern low‐elevation sites, and Group 8 was enriched in Bhutan. Group 7, the largest assigned group, showed greater genetic affinity with Group 6. Groups 1, 4, and 5 were closer to Group 8 and the high‐elevation Group 2, whereas Group 3 occupied an intermediate position among several major groups.

Because several genetic groups contained fewer than 20 accessions, group‐specific LD decay could not be estimated reliably. We therefore used pairwise *F*_st_ to quantify differentiation among the *K* = 8 groups (Figure S4B). Group 7 was strongly differentiated from the other groups, with pairwise *F*_st_ values ranging from 0.510 with Group 6 to 0.763 with Group 2. Groups 1, 4, and 5 were less differentiated from Group 8 than from Group 2, consistent with their intermediate positions in the phylogenetic and ordination analyses. The admixed accessions had comparatively lower *F*_st_ values with most assigned groups.

The maximum‐likelihood tree and PCA were broadly concordant with the ADMIXTURE assignments (Figure 3C,D and Figure S2C–E). Groups 2, 6, and 8 formed relatively coherent genetic assemblages, while Groups 1, 3, 4, and 5 occupied intermediate or partially overlapping positions. Group 7 remained distinct but was positioned closer to Group 6 than to the high‐elevation or Bhutan‐enriched groups. The admixed accessions were dispersed across the tree and PCA space. Together, these analyses indicate that the panel contains both differentiated regional groups and accessions with intermediate or mixed ancestry.

### 3.3 Geographical distribution pattern and phenotypic variations between different subgroups

Geospatial stratification analysis revealed clear geographical differentiation among genetically distinct subpopulations of Himalayan wheat landraces, exhibiting a broad correspondence between geographic distribution, altitudinal gradients, and genetic structure (Figure 4A). Groups 2, 6, and 8 showed contrasting geographical distributions: Group 2 (HA) occupies the northern, high‐altitude mountainous regions; Group 6 (LA) is distributed across the southern low‐altitude valleys and plains; and Group 8 (BT) is predominantly clustered within Bhutan. Crucially, the spatial distribution of the transitional subgroups broadly corresponds to their genetic affinities. Group 7, which showed genetic proximity to the low‐altitude lineage, is primarily concentrated in the lower altitude to mid‐altitude regions, bridging the southern plains. Conversely, the geographically dispersed Groups 1, 4, and 5 span mid‐ to high‐altitude zones, broadly corresponding to their genetic affinities with the high‐altitude (Group 2) and Bhutanese (Group 8) populations.

**FIGURE 4:**
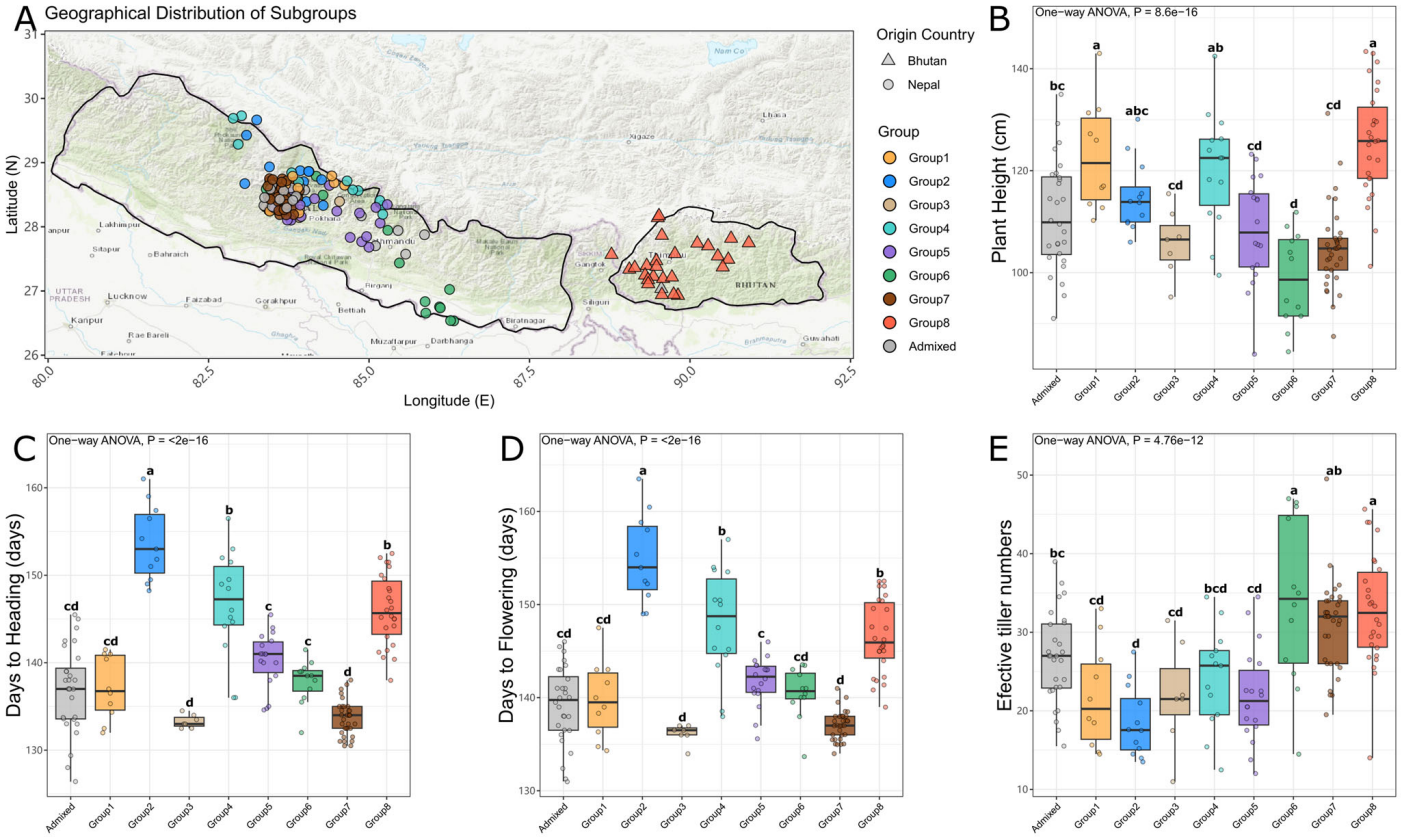
Geographical distribution and phenotypic differences of subpopulations within Himalayan wheat landraces. (A) Geographical distribution of Groups 1–8 and the admixed accessions. (B–E) Variation in plant height, days to heading, days to flowering, and effective tiller number among genetic groups. One‐way analysis of variance (ANOVA) *p* values are shown above each panel. Different lowercase letters indicate significant pairwise differences according to Tukey's honestly significant difference (HSD) test at *p* < 0.05.

To better understand the population history underlying this geographic feature, we measured spatial variation in relative effective migration for the refined panel of 164 Himalayan wheat endemics using FEEMS (Marcus et al., 2021). Given the geographic breadth of our sample collection, we chose two lambda values, 0.2 and 2, to fit the spatial maps (Figure S4A). Both give very similar geographic distribution patterns: severe segregation between high and low elevations and a mix of features in the mountainous areas of western and northwestern Pokhara. Specifically, samples from the Terai Plains region of Nepal and a small number of lowland samples (predominantly corresponding to the low‐altitude Group 6 and its closely related Group 7) are isolated from other subpopulations in the north, creating a mobility barrier that roughly overlaps the Kathmandu–Pokhara line. In contrast, relatively isolated distributions from the high‐altitude (Group 2) and Bhutanese (Group 8) subpopulations can be observed in the northern mountains of Nepal and Bhutan. Notably, many samples from the western and northwestern mountainous regions of Pokhara showed a mixed geographical distribution; however, the effective migration rate in this region was minimal. This suggests that other factors may be impeding gene flow between germplasms.

To determine whether the inferred genetic groups also differed phenotypically, we evaluated PH, DTH, DTF, and ETN under a common growing environment (Figure 4B–E). All four traits differed significantly among groups (one‐way ANOVA, *p* < 0.001), and DTH, DTF, and PH were significantly correlated with collection‐site altitude (Figure S5). Group 2, which occurred predominantly at high‐elevation collection sites, had the latest heading and flowering and the lowest ETN. Group 6, found mainly at lower elevations, was shorter, headed and flowered earlier, and had the highest ETN. Group 8 was the tallest group and combined late flowering with high tillering.

Phenotypic patterns also broadly corresponded to genetic affinities. Group 7 resembled Group 6 in shorter PH, earlier phenology, and higher ETN. Groups 1, 4, and 5 generally showed intermediate to taller PH and later heading and flowering. In contrast, Group 3 and the admixed accessions had broad, intermediate trait distributions. These results show that the genetic groups also capture agro‐morphological differentiation associated with collection geography and elevation. Detailed subgroup distributions and regional shares are provided in Figure S6.

### 3.4 Relationship between the Himalayan wheat landrace panel and the global hexaploid wheat core collection

Integration of Himalayan germplasms into the hexaploid core collection by Takenaka et al. (2019) enabled comparative localization of these accessions within global wheat diversity using shared molecular marker datasets (see Table S2). Using the same filtered marker set, the NBRP global core panel had higher genome‐wide nucleotide diversity (*π* = 0.2286), Watterson's theta (*θ* = 0.1595), expected heterozygosity (*H_e_* = 0.0930), and PIC (= 0.0831) than the Himalayan panel (*π* = 0.0666, *θ* = 0.0856, *H_e_* = 0.0821, and PIC = 0.0734; Table 1). LD decay was also estimated separately for the two panels using the same analytical procedure. The fitted LD curve reached half of its maximum at 3.71 and 3.09 Mbp, *r* ^2^ = 0.2 at 4.79 and 3.98 Mbp, and *r* ^2^ = 0.1 at 13.75 and 11.45 Mbp in the Himalayan and NBRP global core panels, respectively (Figure S7C). These comparisons show that the Himalayan panel does not exceed the global core panel in overall marker‐based diversity and exhibits modestly more extended LD.

Population structure analysis revealed a broad differentiation of Himalayan germplasm from global counterparts at *K* = 3. In contrast, the minimum CV error occurred at *K* = 14 (Figure 5A and Figure S7A,B). Phylogenetic reconstruction demonstrated close evolutionary relationships between Himalayan accessions and outlier samples from East Asia (Tibet of China), South Asia, and the Middle East (Figure 5B). Bhutanese samples formed a monophyletic cluster embedded within Nepalese accessions, consistent with previous findings.

**FIGURE 5:**
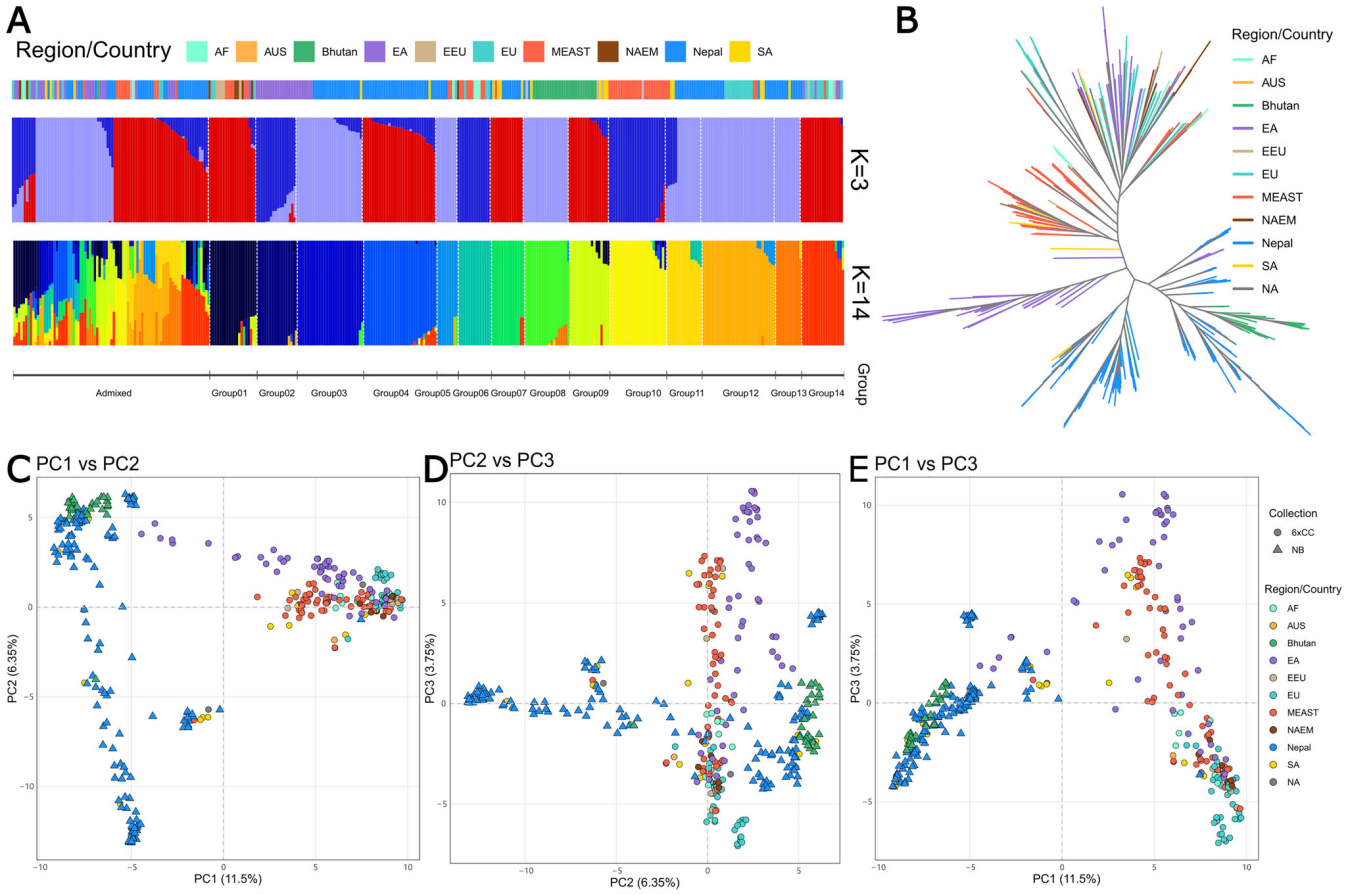
Genetic relationship of the Himalayan wheat landraces and the hexaploid wheat core collection. (A) Population structure inferred from pruned markers with ADMIXTURE, at *K* = 3 and *K* = 14; (B) Maximum likelihood phylogenetic tree, branch colored by geographical origins. (C–E) Principal component analysis (PCA) scores based on pruned markers, colored by geographical origins.

The top three principal components from PCA further elucidated genetic relationships (Figure 5C–E and Figure S7D). Himalayan germplasm exhibited tight clustering along the PC1 and PC3 axes, whereas it showed marked dispersion along PC2. Bhutanese accessions (green triangles) clustered proximally while being phylogenetically nested within Nepalese samples. Figure 5C–E revealed genetic continuity between Himalayan accessions and those from East Asia, South Asia, and the Middle East. Group 7 extended strongly along PC2, while Group 8 and several genetically intermediate accessions also occupied areas with limited overlap with the evaluated global core accessions. (Figure 5C,D). These results indicate that the Himalayan panel contributes complementary regional variation rather than greater overall marker‐based diversity.

## 4 DISCUSSION

### 4.1 Genetic diversity of the Himalayan wheat landrace panel in a global context

Large‐scale wheat landrace collections have provided broad descriptions of global population structure and have demonstrated the value of historical germplasm for crop improvement (Cheng et al., 2024; Wingen et al., 2014). Such panels, however, necessarily sample individual regions at relatively low density and may therefore overlook differentiation within geographically complex areas. The present Himalayan panel provides finer regional resolution across Nepal and Bhutan, spanning collection sites from 400 to 3900 m in elevation. At this scale, the accessions do not form a homogeneous Himalayan gene pool. Instead, they comprise differentiated genetic groups, together with transitional and admixed accessions distributed across contrasting geographical and elevational settings. This internal structure, rather than unusually high diversity across the panel, is its main distinguishing feature.

Across the filtered marker set, the refined Himalayan panel displayed lower nucleotide diversity (*π* = 0.0666) and Watterson's theta (*θ* = 0.0856), alongside lower expected heterozygosity (*H_e_* = 0.0821) and PIC (0.0734), relative to the globally sourced NBRP core collection (*π* = 0.2286, *θ* = 0.1595, *H_e_* = 0.0930, and PIC = 0.0831), which is consistent with the core panel's much broader geographical provenance. On the other hand, LD was modestly more extended in the Himalayan panel, with the fitted curve reaching *r* ^2^ = 0.2 at 4.79 Mbp, compared with 3.98 Mbp in the NBRP global core panel (Figure S7C). These results show that the Himalayan collection does not exceed the global collection in overall marker‐based diversity but may reflect variation in population composition, demographic history, and internal population structure (Chao et al., 2010; Roncallo et al., 2021). Subgenome‐level analysis revealed consistently lower diversity in the Himalayan panel across the A and B subgenomes. In both panels, the D subgenome displayed lower *H_e_* and PIC values than the A and B subgenomes, consistent with the well‐documented genetic bottleneck during the formation of hexaploid wheat from its wild progenitor *Aegilops tauschii* (The International Wheat Genome Sequencing Consortium (IWGSC) et al., 2018).

Despite lower overall genetic diversity than the global hexaploid wheat core collection (as evidenced by the overall *H_e_* and PIC in Table 1), the population structure analysis revealed distinct genetic clusters within the Himalayan panel. Although the NBRP core panel contains 10 Himalayan accessions (6 × 076–6 × 085), principal component and phylogenetic analyses revealed that Group 7, Group 8 (enriched in Bhutan), and several transitional accessions occupy regions of genetic space with limited representation from the evaluated core panel. High pairwise *F*_st_ values (Figure S4B) also support genetic differentiation among these groups within the Himalayan panel. Specifically, Group 7 exhibits notable genetic divergence from other subgroups, with strong differentiation from the high‐altitude Group 2 (*F*_st _= 0.763) and the Bhutanese Group 8 (*F*_st _= 0.631). Similarly, Group 8 remains strongly differentiated from the northern high‐altitude lineages (*F*_st _= 0.599 with Group 2). In the PCA, these groups extended along the PC2 axis into regions with limited overlap with accessions from the evaluated core panel. These comparisons show that genetic variation within Nepal and Bhutan is more strongly structured than the limited number of Himalayan accessions in the global core panel would suggest.

The limited representation of Nepalese and Bhutanese wheat in the evaluated core panel reflects a broader regional sampling gap in historical germplasm resources. For example, the Watkins collection—comprising over 800 wheat landraces and serving as a foundational resource for diversity studies—includes limited representation from Nepal and Bhutan, particularly their high‐elevation regions (Cheng et al., 2024; Wingen et al., 2014). The genetically differentiated accessions identified here therefore add regional diversity that is poorly represented in the evaluated core panel and merit further phenotypic evaluation, allele discovery, and pre‐breeding.

### 4.2 Population structure reflects regional geography and historical gene flow

The eight genetic groups and the additional admixed category showed clear geographical patterning across the sampled region. Group 2 occurred predominantly in northern high‐elevation areas, Group 6 in southern low‐elevation areas, and Group 8 was concentrated in Bhutan. In contrast, transitional and admixed accessions were distributed across intervening regions. All four agro‐morphological traits also differed among groups in a common growing environment, indicating that the genetic structure coincided with phenotypic and geographical differentiation. We therefore interpret these groups as regional genetic assemblages rather than discrete ecotypes. Their distribution may reflect multiple, partly overlapping processes, including topographic separation, historical crop movement, and farmer‐mediated seed exchange.

FEEMS identified an area of reduced effective migration between the regions occupied predominantly by Groups 2 and 6. This spatial discontinuity broadly coincided with steep elevational transitions and mountainous terrain, suggesting that topography may have restricted movement among regional seed populations more strongly than geographical distance alone (Funk et al., 2016). However, FEEMS estimates relative effective migration from spatial patterns of genetic dissimilarity and does not identify the underlying cause of a migration boundary. Transitional and admixed accessions were concentrated in central mountain valleys, including areas surrounding Pokhara and Kathmandu, which may have served as contact zones among regional populations. Farmer‐mediated seed exchange, local trade routes, and associated cultural practices provide plausible routes for such connectivity (Wu et al., 2022). However, the present data cannot distinguish their contributions from older demographic history or shared ancestry.

The broader geographical pattern is also consistent with archaeobotanical and historical evidence that the Himalayas formed an agricultural contact zone between the Indo‐Gangetic plains to the south and the Qinghai–Tibetan Plateau to the north (C.‐C. Liu et al., 2022; Wu et al., 2022). Our genetic and phenotypic data are consistent with this history of agricultural exchange (Figure 4B–E). Group 6 and the genetically proximate Group 7, both distributed mainly across lower elevation to mid‐elevation regions, were characterized by shorter PH, earlier heading and flowering, and higher ETN. By contrast, Group 2, Group 8, and the transitional Groups 1, 4, and 5 were associated mainly with mid‐ to high‐elevation regions and generally displayed greater PH and later heading and flowering. These correspondences show that genetic structure covaries with collection geography and phenotype, but they do not resolve the direction or timing of historical crop movement. Connections with southern and northern agricultural traditions therefore remain plausible historical interpretations that require broader regional sampling and formal demographic modeling.

Group 8 illustrates why elevation alone cannot explain the observed differentiation. Although Group 8 originated predominantly from relatively high‐elevation sites and showed genetic affinity with Group 2, it had significantly higher ETN than Group 2 and did not differ significantly from Groups 6 and 7 in this trait (Figure 4E). This result contrasts with earlier general descriptions of traditional Bhutanese wheat as tall, weak‐stemmed, and poorly tillering (Mann & Hobbs, 1988). Differences in accession sampling, growing conditions, and local management history could explain the contrast between studies. Mann and Hobbs (1988) also reported that up to 50% of wheat grown at mid‐ to high elevations was traditionally harvested green for animal fodder. This practice raises the possibility that farmer selection for vegetative biomass or tillering influenced some Bhutanese landraces. The present data, however, neither quantify historical selection nor directly connect this management practice to the phenotype of Group 8, and this explanation should therefore be treated as a hypothesis for future evaluation.

Taken together, the regional differentiation, transitional groups, and extensive admixture are consistent with the combined influence of topography, population history, and human movement rather than with a single model of geographical isolation. The phenotypic differences observed in a common environment show that the genetic groups also capture agro‐morphological differentiation. However, associations among population structure, phenotype, and collection‐site altitude do not, by themselves, demonstrate local adaptation. Collection‐site altitude summarizes a complex environmental gradient but does not identify the climatic or edaphic factors underlying these associations. Multi‐environment trials incorporating explicit environmental covariates and genotype‐by‐environment (G × E) analyses will be required to test adaptive hypotheses; subsequent genome‐wide association and environmental association analyses (EAA) could then identify associated loci.

### 4.3 Historical significance and prospective breeding value of the Himalayan collection

Landraces serve as foundational resources in crop breeding, offering novel alleles and acting as intermediates for adapting elite germplasm to diverse agroecological conditions (Cheng et al., 2024). The Himalayan collection provides a historically documented record of regional wheat germplasm maintained before the widespread adoption of Green Revolution cultivars. The differentiated groups identified here provide a basis for prioritizing accessions for further phenotypic and genomic evaluation.

A defining feature of this panel is its collection period, 1952–1958. Nepal's national wheat program began in 1951. However, its first improved cultivar, Lerma 52, was not released until 1960 (Khadka et al., 2020; Morris et al., 1994), while comparable breeding initiatives in India and the Qinghai–Tibetan Plateau expanded during the 1960s (Eliazer Nelson et al., 2019; He, 2001). The panel therefore provides a historically dated record of wheat germplasm maintained at the sampled locations before the widespread regional adoption of Green Revolution cultivars. However, because this study did not include matched modern cultivars or contemporary collections from the same localities, it cannot quantify temporal changes in diversity or demonstrate genetic erosion. Furthermore, the historical expeditions followed specific collection routes and likely missed isolated valleys and microenvironments. The panel should therefore be regarded as a geographically broad but incomplete historical sample of Nepalese and Bhutanese wheat germplasm.

Previous studies have highlighted the risk of genetic contamination in germplasm collected post‐Green Revolution. In Nepal, for example, spring wheat samples obtained in the 1970s and beyond may include exotic varieties that were misidentified as traditional landraces due to decades of farmer adoption (Khadka et al., 2020). Therefore, the earlier collection date reduces the likelihood that these accessions represent cultivars introduced during the subsequent expansion of modern breeding programs. Nevertheless, collection date alone does not demonstrate genetic replacement or loss, which would require direct comparison with well‐documented contemporary germplasm sampled from comparable locations.

The historical context of the collection is also relevant to interpreting its regional differentiation. The landraces in this panel are products of ancient agricultural systems situated at the intersection of two major centers of wheat cultivation. Archaeobotanical evidence indicates that wheat was cultivated in the Indian subcontinent as early as 4000–2000 BCE (Tengberg, 1999) and on the Qinghai–Tibetan Plateau by at least 1400 BCE (F. H. Chen et al., 2015; d'Alpoim Guedes et al., 2015), having reached northern China around 2600 BCE (Long et al., 2018). At the intersection of these agricultural regions, the genetic differentiation of Himalayan landraces may reflect the combined influence of topography, population history, and human‐mediated crop movement. However, its adaptive significance requires direct evaluation.

In this study, we use GRAS‐Di for genotyping. It is a highly cost‐effective strategy that effectively captures pericentromeric regions and provides reliable genotypic resolution for macro‐level population‐structure mapping and broad diversity assessments (Hosoya et al., 2019; Su et al., 2026). However, compared to whole‐genome sequencing (WGS) or high‐density SNP arrays, GRAS‐Di inherently suffers from certain limitations, including lower overall marker density and higher rates of missing data. Consequently, while the current GRAS‐Di dataset is highly adequate for elucidating the phylogeography and population structure presented here, future studies aiming to pinpoint rare functional variants will require WGS.

## 5 CONCLUSIONS AND FUTURE PERSPECTIVES

This study provides a genomic and agro‐morphological characterization of a historically documented wheat landrace collection from Nepal and Bhutan. After removing close genetic duplicates, 164 accessions formed eight geographically patterned genetic groups and an additional admixed category. The groups differed in four traits measured in a common environment. The coexistence of differentiated, transitional, and admixed accessions was inconsistent with a model of simple geographic isolation. Although the Himalayan panel had lower overall marker‐based diversity than the NBRP global core panel, Group 7, the Bhutan‐enriched Group 8, and several transitional accessions occupied regions of genetic space with limited representation in the evaluated global core panel. This Himalayan panel provides fine‐scale regional representation of a historically sampled Himalayan gene pool and a basis for prioritizing accessions for further genomic and phenotypic evaluation.

Building on these insights, several key directions for future research are evident. First, while the GRAS‐Di platform provided robust genotypic resolution, it is not designed to detect large structural variations (SVs). Therefore, future WGS of a representative subset would enable finer‐scale detection of genetic variation. Furthermore, existing high‐quality wheat reference genomes (such as Chinese Spring) represent genetic backgrounds that evolved outside extreme Himalayan environments. We hypothesize that extreme high‐altitude adaptation may be driven by unique alleles or complex SVs (e.g., large inversions or copy‐number variants) that are simply absent from current reference genomes.

Second, extensive phenotypic characterization is essential to test hypotheses of high‐altitude adaptation and investigate their genetic basis. Multi‐location, multi‐year field trials—particularly those spanning altitudinal gradients—are needed to dissect G × E interactions. Under these conditions, the evaluation of an extended suite of traits, including growth and yield parameters, resistance to abiotic and biotic stresses, and grain quality attributes (e.g., grain morphology, macronutrient composition, and micronutrient density), will be critical. Evaluating these grain quality parameters is highly relevant to local farming systems, as Himalayan landraces are often valued by farmers for specific culinary traditions and potential nutritional benefits, which play an important role in regional nutritional security and local market acceptance. Ultimately, integrating these robust multi‐environment phenotypic datasets and explicit environmental covariates with genotypic data to perform genome‐wide association studies and EAA could help identify candidate adaptation‐related loci for subsequent validation.

Finally, while this study identifies the raw genetic resource and structural framework of these landraces, translating these findings into practical breeding applications requires substantial pre‐breeding efforts, including the identification and functional validation of specific markers. The development of pre‐breeding populations, such as Nested Association Mapping (NAM) or Multi‐parent Advanced Generation Inter‐Cross (MAGIC) populations, represents a strategic next step. However, the direct utilization of these diverse Himalayan landraces in breeding programs is highly constrained by linkage drag and poor agronomic adaptation (e.g., lodging due to extremely tall stature). Therefore, careful pre‐breeding strategies, including targeted introgression and extensive backcrossing, are necessary to overcome these barriers. By crossing genetically diverse Himalayan landraces with regionally adapted elite cultivars, it will be possible to generate powerful resources for fine‐mapping adaptive loci and accelerating the development of improved wheat varieties tailored to the needs of local Himalayan farmers.

Beyond breeding applications, our findings highlight the critical importance of conservation genetics. Because we did not analyze a matched contemporary population, this study does not establish the extent of genetic erosion in Nepal or Bhutan. Instead, the NBRP collection provides a historically dated ex situ resource that can support future temporal comparisons with contemporary on‐farm populations sampled from comparable locations. To dynamically preserve this unique evolutionary heritage, there is an urgent need for a synergistic approach combining ex situ conservation (e.g., in genebanks) with in situ conservation (on‐farm management by local communities). This collection provides a documented set of genetically differentiated accessions that can be prioritized for future evaluation under defined climate‐relevant stress conditions. Any contribution to climate‐resilience breeding will need to be established through multi‐environment phenotyping and functional validation of candidate loci. The recent identification of two potentially novel yellow rust resistance loci within this panel (Jung et al., 2025) provides a rationale for further evaluation; however, these loci currently remain putative associations and await further independent functional validation to facilitate their use in breeding.

## AUTHOR CONTRIBUTIONS

**Li Yunfei**: Conceptualization; formal analysis; visualization; writing—original draft; writing—review and editing. **Meng Zhaojie**: Formal analysis; visualization; writing ‐ original draft; writing—review and editing. **Miyuki Nitta**: Data curation; investigation; resources. **Atsushi Ohta**: Data curation; investigation; resources. **Kazuhiro Nemoto**: Data curation; investigation. **Giri Prasad Joshi**: Data curation; investigation. **Su Zhuo**: Formal analysis; writing—review and editing. **Yasir Serag Alnor Gorafi**: Investigation; supervision; writing—review and editing. **Shuhei Nasuda**: Conceptualization; funding acquisition; methodology; project administration; resources; supervision; writing—review and editing.

## CONFLICT OF INTEREST STATEMENT

The authors declare no conflicts of interest.

## Supporting information




**Supplemental Figure S1. Duplicate identification through pairwise‐DST values**. (A) Distribution of pairwise identical‐by‐state (IBS) similarity (DST) values among the initial 202 accessions (zoomed in for DST > 0.95). The vertical dashed orange line indicates the threshold of 0.98. (B) The top 500 pairwise DST values ranked in descending order. The horizontal dashed red line represents the stringent cutoff of 0.98, which was applied to identify and remove 38 duplicated accessions.
**Supplemental Figure S2. Population structure and principal component analysis of Himalayan wheat landraces**. (A) Population structure estimated using ADMIXTURE across the evaluated values of K. The upper annotation bars indicate collection‐site elevation and country of origin for each accession. (B) CV error across the evaluated values of K. Although the minimum CV error occurred at *K* = 10, *K* = 8 was the first local minimum and was selected as the parsimonious grouping level for downstream analyses. At *K* = 8, accessions with *Q*_max_ ≥ 0.70 were assigned to the corresponding genetic group, whereas those with *Q*_max_ < 0.70 were classified as admixed. (C–E) Principal component analysis plots showing PC1 versus PC2, PC1 versus PC3, and PC2 versus PC3 based on 5,573 LD‐pruned markers. Accessions are colored according to their K = 8 group assignments and shaped by country of origin.
**Supplemental Figure S3. Pairwise genetic distance matrix of Himalayan wheat landraces**. The heatmap visualizes the genetic distance among the 164 unique accessions. The upper and left dendrograms were constructed using hierarchical clustering. Annotation bars on the top and right indicate the assigned genetic subgroup (Group 1–8 and Admixed), collection site altitude, and regional/country origin for each accession.
**Supplemental Figure S4. Spatial variation in relative effective migration and genetic differentiation among Himalayan wheat landraces**. (A) Relative effective migration surfaces inferred using fast estimation of effective migration surfaces (FEEMS) for the 164 retained accessions, with smoothing parameter λ = 2.0 (upper panel) and λ = 0.2 (lower panel). Edge colors indicate inferred relative effective migration rates, with orange indicating lower rates and blue indicating higher rates. Circle sizes represent the numbers of accessions assigned to sampled nodes. Larger λ values impose stronger spatial smoothing. These surfaces summarize spatial patterns of genetic dissimilarity and do not directly measure contemporary seed movement or identify the causes of inferred migration differences. (B) Pairwise fixation index (*F*_st_) estimates among Groups 1–8 and the admixed category. Cell labels give the estimated *F*_st_ values, with warmer colors indicating greater genetic differentiation. The admixed category comprises accessions with a maximum ancestry proportion below 0.70 at K = 8 and does not represent an additional ancestral population.
**Supplemental Figure S5. Correlation between collection site altitude and agro‐morphological traits of Himalayan wheat landraces**. (A) Geographic map showing the altitudes of each collection site for the refined accessions. (B) Spearman correlation matrix evaluating the relationships between altitude, plant height (PH), days to heading (DTH), days to flowering (DTF), and effective tiller number (ETN). Asterisks denote statistical significance (** *p* < 0.01, *** *p* < 0.001). (C–F) Geographical distribution and phenotypic variations of DTH, DTF, PH, and ETN.
**Supplemental Figure S6. Detailed geographical distribution and regional shares of subgroups within the Himalayan wheat collection**. (A) Individual distribution maps for the eight assigned subgroups (Group 1–8), the Admixed group, and the identified duplicated accessions (Dup) across the initial 202 collected samples. For each map, pie charts represent the percentage proportion of the specific subgroup relative to the total initial collection. (B) A comprehensive spatial map depicting the precise geographic coordinates of all 164 retained Himalayan wheat landraces, colored by their assigned genetic subgroups.
**Supplemental Figure S7. Population structure, linkage disequilibrium (LD) decay, and PCA of the combined Himalayan landraces and hexaploid core collection panel**. (A) CV error of different K values from 10 cross‐validation runs on ADMIXTURE, identifying best K = 14. (B) Population structure estimated with ADMIXTURE at representative K values (K = 3, 5, 7, 9, 11–14). (C) Linkage disequilibrium decay estimated separately for the Himalayan panel (n = 164) and the NBRP global hexaploid wheat core panel (n = 187). Curves were fitted to all valid pairwise LD estimates using the Hill–Weir nonlinear model. Background points represent distance‐stratified random subsamples used only for visualization. Symbols indicate the fitted distances at half‐decay, r^2^ = 0.2, and r^2^ = 0.1. (D) 3D PCA plot showing the genetic relationship between the Himalayan landrace collection and the globally diverse hexaploid wheat core collection, colored by geographical regions.

**Supplemental Table S1**. Accession number and passport information of Himalayan wheat accessions in NBRP‐wheat.
**Supplemental Table S2**. Accession number and passport information of NBRP hexaploid wheat core collection (Derived from Takenaka et al., 2019).
**Supplemental Table S3**. Statistics of quality‐controlled clean reads of NBRP Himalayan wheat and hexaploid core collection.
**Supplemental Table S4**. Genetic principal components, subpopulation stratification, and phenotype data of Himalayan wheat landraces.

## Data Availability

The genotypic data (GRAS‐Di raw reads and derived variant calling files) are available from the corresponding author upon reasonable request.
