# Supplementary material for: Genomic characterization of pre‐Green Revolution wheat landraces from the Nepalese and Bhutanese Himalayas reveals geographically structured diversity
Source: Plant Genome. 2026 Oct 12;19(4):e70320. doi: 10.1002/tpg2.70320 (PMC13658851; doi:10.1002/tpg2.70320)
Supplement: Supplementary file 1 [file TPG2-19-e70320-s001.docx]

Genomic characterization of pre-Green Revolution wheat landraces from the Nepalese and Bhutanese Himalayas reveals geographically structured diversity

Li Yunfei^1^, Meng Zhaojie^1^, Miyuki Nitta^1^, Atsushi Ohta^1^, Kazuhiro Nemoto^2^, Giri Prasad Joshi^3^, Su Zhuo^1^, Yasir Serag Alnor Gorafi^1^, Shuhei Nasuda^1*^

Affiliations:

1. Graduate School of Agriculture, Kyoto University, Kitashirakawaoiwake-cho, Sakyo-ku, Kyoto-shi, Kyoto 6060-8502 Japan

2. Faculty of Agriculture, Shinshu University, 8304 Minamiminowa-mura, Kamiina-gun, Nagano 399-4598 Japan

3. Central Department of Botany, Tribhuvan University, Kirtipur, Kathmandu 44600 Nepal

Supplementary Tables

**Supplemental Table S1 (In a separate spread sheet file).** Accession number and passport information of Himalayan wheat accessions in NBRP-wheat.

**Supplemental Table S2 (In a separate spread sheet file).** Accession number and passport information of NBRP hexaploid wheat core collection (Derived from Takenaka et. al., 2019).

**Supplemental Table S3 (In a separate spread sheet file).** Statistics of quality-controlled clean reads of NBRP Himalayan wheat and hexaploid core collection.

**Supplemental Table S4 (In a separate spread sheet file).** Genetic principal components, subpopulation stratification, and phenotype data of Himalayan wheat landraces.

Supplementary figures

**Supplemental Figure S1. Duplicate identification through pairwise-DST values.** (A) Distribution of pairwise identical-by-state (IBS) similarity (DST) values among the initial 202 accessions (zoomed in for DST > 0.95). The vertical dashed orange line indicates the threshold of 0.98. (B) The top 500 pairwise DST values ranked in descending order. The horizontal dashed red line represents the stringent cutoff of 0.98, which was applied to identify and remove 38 duplicated accessions.

**Supplemental Figure S2. Population structure and principal component analysis of Himalayan wheat landraces.** (A) Population structure estimated using ADMIXTURE across the evaluated values of K. The upper annotation bars indicate collection-site elevation and country of origin for each accession. (B) Cross-validation error across the evaluated values of K. Although the minimum cross-validation error occurred at K = 10, K = 8 was the first local minimum and was selected as the parsimonious grouping level for downstream analyses. At K = 8, accessions with *Qmax* ≥ 0.70 were assigned to the corresponding genetic group, whereas those with *Qmax* < 0.70 were classified as admixed. (C–E) Principal component analysis plots showing PC1 versus PC2, PC1 versus PC3, and PC2 versus PC3 based on 5,573 LD-pruned markers. Accessions are colored by K = 8 group assignments and shaped by country of origin.

**Supplemental Figure S3. Pairwise genetic distance matrix of Himalayan wheat landraces.** The heatmap visualizes the genetic distance among the 164 unique accessions. The upper and left dendrograms were constructed using hierarchical clustering. Annotation bars at the top and right indicate the assigned genetic subgroup (Group 1–8 and Admixed), collection site altitude, and regional/country origin for each accession.

**Supplemental Figure S4.** **Spatial variation in relative effective migration and genetic differentiation among Himalayan wheat landraces.** (A) Relative effective migration surfaces inferred using fast estimation of effective migration surfaces (FEEMS) for the 164 retained accessions, with smoothing parameter λ = 2.0 (upper panel) and λ = 0.2 (lower panel). Edge colors indicate inferred relative effective migration rates, with orange indicating lower rates and blue indicating higher rates. Circle sizes represent the numbers of accessions assigned to sampled nodes. Larger λ values impose stronger spatial smoothing. These surfaces summarize spatial patterns of genetic dissimilarity and do not directly measure contemporary seed movement or identify the causes of inferred migration differences. (B) Pairwise fixation index (*F_st_*) estimates among Groups 1–8 and the admixed category. Cell labels give the estimated *F_st_* values, with warmer colors indicating greater genetic differentiation. The admixed category comprises accessions with a maximum ancestry proportion below 0.70 at K = 8 and does not represent an additional ancestral population.

**Supplemental Figure S5. Correlation between collection site altitude and agro-morphological traits of Himalayan wheat landraces.** (A) Geographic map showing the altitude for each collection site of the refined accessions. (B) Spearman correlation matrix evaluating the relationships between altitude, plant height (PH), days to heading (DTH), days to flowering (DTF), and effective tiller number (ETN). Asterisks denote statistical significance (** p < 0.01, *** p < 0.001). (C–F) Geographical distribution and phenotypic variations of DTH, DTF, PH, and ETN.

**Supplemental Figure S6. Detailed geographical distribution and regional shares of subgroups within the Himalayan wheat collection.** (A) Individual distribution maps for the eight assigned subgroups (Groups 1–8), the Admixed group, and the identified duplicated accessions (Dup) across the initial 202 collected samples. For each map, pie charts represent the percentage proportion of the specific subgroup relative to the total initial collection. (B) A comprehensive spatial map showing the reported geographic coordinates of all 164 retained Himalayan wheat landraces, colored by their assigned genetic subgroups.

**Supplemental Figure S7. Population structure, linkage disequilibrium (LD) decay, and PCA of the combined Himalayan landraces and hexaploid core collection panel.** (A) CV error of different K values from 10 cross-validation runs on ADMIXTURE, identifying Best K=14. (B) Population structure estimated with Admixture at representative K values (K=3, 5, 7, 9, 11–14). (C) Linkage disequilibrium decay estimated separately for the Himalayan panel (n = 164) and the NBRP global hexaploid wheat core panel (n = 187). Curves were fitted to all valid pairwise LD estimates using the Hill–Weir nonlinear model. Background points represent distance-stratified random subsamples used for visualization only. Symbols indicate the fitted distances at half-decay, *r²* = 0.2, and *r²* = 0.1. (D) 3D PCA plot showing the genetic relationship between the Himalayan landrace collection and the globally diverse hexaploid wheat core collection, colored by geographical regions.
